# *Anopheles subpictus* carry human malaria parasites in an urban area of Western India and may facilitate perennial malaria transmission

**DOI:** 10.1186/s12936-016-1177-x

**Published:** 2016-02-27

**Authors:** Ashwani Kumar, Rajeshwari Hosmani, Shivaji Jadhav, Trelita de Sousa, Ajeet Mohanty, Milind Naik, Adarsh Shettigar, Satyajit Kale, Neena Valecha, Laura Chery, Pradipsinh K. Rathod

**Affiliations:** National Institute of Malaria Research, Field Unit, Campal, Goa, India; National Institute of Malaria Research (ICMR), Sector 8, Dwarka, New Delhi, India; Department of Chemistry, University of Washington, Seattle, WA USA

**Keywords:** Malaria, Goa, New vector, Salivary glands, *Plasmodium*, *Anopheles subpictus*

## Abstract

**Background:**

India contributes 1.5–2 million annual confirmed cases of malaria. Since both parasites and vectors are evolving rapidly, updated information on parasite prevalence in mosquitoes is important for vector management and disease control. Possible new vector-parasite interactions in Goa, India were tested.

**Methods:**

A total of 1036 CDC traps were placed at four malaria endemic foci in Goa, India from May 2013 to April 2015. These captured 23,782 mosquitoes, of which there were 1375 female anopheline specimens with ten species identified using morphological keys. Mosquito DNA was analysed for human and bovine blood as well as for *Plasmodium falciparum* and *Plasmodium vivax* infection.

**Results:**

Human host feeding was confirmed in *Anopheles stephensi* (30 %), *Anopheles subpictus* (27 %)*, Anopheles jamesii* (22 %), *Anopheles annularis* (26 %), and *Anopheles nigerrimus* (16 %). In contrast, *Anopheles vagus*, *Anopheles barbirostris*, *Anopheles tessellates, Anopheles umbrosus* and *Anopheles karwari* specimens were negative for human blood. Importantly, *An. subpictus*, which was considered a non-vector in Goa and Western India, was found to be a dominant vector in terms of both total number of mosquitoes collected as well as *Plasmodium* carriage. *Plasmodium* infections were detected in 14 *An. subpictus* (2.8 %), while the traditional vector, *An. stephensi,* showed seven total infections, two of which were in the salivary glands. Of the 14 *An. subpictus* infections, nested PCR demonstrated three *Plasmodium* infections in the salivary glands: one *P. vivax* and two mixed infections of *P. falciparum* and *P. vivax.* In addition, ten gut infections (one *P. vivax*, six *P. falciparum* and three mixed infections) were seen in *An. subpictus*. Longitudinal mosquito collections pointed to a bimodal annual appearance of *An. subpictus* to maintain a perennial malaria transmission cycle of both *P. vivax* and *P. falciparum* in Goa.

## Background

India contributes significantly to the total malaria burden in the world with about 1.5–2 million confirmed malaria cases and annual death estimates ranging from 15,000 to 95,000 [1, 2]. The epidemiology of malaria in India is complex due to the geo-ecological diversity of the country, multi-ethnicity of human hosts, wide distribution of different anopheline species, and extensive human mobility within the country and from outside [1]. Historically, the 1950s malaria eradication programme in India, led by World Health Organization (WHO) and focused on vector control using indoor residual insecticide DDT, was considered a success. Malaria incidence in humans decreased from about 75 million in 1947 to less than 100,000 in 1964 [1]. Malaria was then thought to be on the verge of eradication in India, but it re-emerged in 1976 [3].

Areas with unstable malaria present an important opportunity to re-examine human-parasite-vector interactions that contribute to the transmission of malaria. Traditionally, there are six recognized primary vectors of malaria in India, viz., *Anopheles culicifacies*, *Anopheles stephensi*, *Anopheles dirus* (*Anopheles baimaii*), *Anopheles fluviatilis*, *Anopheles minimus* and *Anopheles sundaicus*. Vectors of secondary importance are *Anopheles annularis*, *Anopheles varuna*, *Anopheles jeyporiensis* and *Anopheles philippinensis* [4]. *Anopheles stephensi* has been incriminated repeatedly and strongly in different parts of India. The species is capable of transmitting malaria at very low densities and is an important urban malaria vector in the country [4].

In the coastal belt of Goa, following a 1986 malaria outbreak in urban Panaji, *An. stephensi* was extensively studied for its role as the primary vector in malaria transmission in terms of both its resting and biting behaviour and its breeding habits [5–8]. A recent study carried out in Goa showed that one out of 54 *An. stephensi* was positive for *P. falciparum* malaria infection with CSP ELISA [9]. The prevailing view has been that other anopheline species do not contribute significantly to malaria transmission along the Western Coast, including Goa. In addition to *An. stephensi* in urban areas, Goa also has at least two other recognized rural malaria vectors, *An. culicifacies* and *An. fluviatilis* [8, 10]. *Anopheles culicifacies* is found in low densities in the sub-coastal belt of Goa where it breeds in irrigation channels, ponds and rice paddies and *An. fluviatilis* is abundant in the eastern hilly regions of the state where it breeds in stagnant waters in streams and paddy fields [8, 10]. There have been reports of *An. dirus* in forested hinterlands of Goa, but it has not been extensively studied for its role in malaria transmission [11].

The present study involved a broad and unbiased re-assessment of vectorial capacity of all significant *Anopheles* mosquitoes in urban Goa. This was motivated, in part, by the MESA-ICEMR hypothesis that parasites and mosquitoes may change their relationships as each comes under increasing pressure from malaria control programs [13, 14]. Other basic science studies show that parasite populations have powerful strategies to select for beneficial changes in their haploid genome without collateral damage [12, 14, 15]. While this has previously been of interest with respect to acquisition of resistance to anti-malarials, it is possible that interactions between parasites and vectors may also be fluid. Unproductive interactions between human malaria parasites and non-vector anophelines may evolve so that human malaria parasites develop successfully in *Anopheles* mosquitoes not suspected to be productive hosts [16]. Continual broad investigation into the role of local anopheline species in malaria transmission areas was, thus, considered important.

Here, nested PCR was employed and both *Plasmodium falciparum* and *P. vivax* were detected in the salivary glands and guts of wild anopheline mosquitoes collected from Goa. The results show that anophelines other than *An. stephensi* may participate in the propagation of human malaria in urban Goa and that parasites may exploit multiple vectors to maintain perennial transmission in Goa. The findings and this particular open-ended approach may have important implications for vector control strategies in urban India, particularly in Goa.

## Methods

### Study area and time

Goa, a state covering a geographical area of 3702 km^2^, is situated on the West Coast of India. It has a population of 1.5 million residing in two districts (North and South Goa) [8]. Construction sites located in Panjim, Candolim, Porvorim (North Goa) and Margao (South Goa) were identified as focal points for the malaria transmission study based on data received from Goa Directorate of Health Services, local Urban and Primary Health Centres, and malaria patient data from MESA-ICEMR operations at Goa Medical College and Hospital. Such targeted deployment of CDC traps maximised the possibility of capturing malaria vectors within active transmission zones. This paper analyses the human and bovine blood meal and the presence of human *Plasmodia* in the mosquitoes collected from May 2013–April 2015 in Goa.

### Mosquito collection

CDC traps (John W. Hock, USA) with UV lights and 6 V batteries were used for trapping mosquitoes. Verbal informed consent was obtained from construction-site engineers and from house owners prior to trap deployment. People residing around the collection sites were informed of the study and they helped protect the traps from disturbance. Traps were hung inside labourer’s shelters at 6 pm and collected at 9 am the following morning, avoiding shelters where firewood was used for cooking. The shelters where collections took place were not sprayed with any insecticides.

### Identification and preservation of mosquitoes

Following deployment and recovery of traps, the mouth of each collection bag was secured to prevent escape of mosquitoes. The bags were separated from trap machines and transported to the NIMR-Goa laboratory with care to avoid damage to the mosquitoes.

Back at the NIMR-Goa lab, the freshly collected mosquitoes were anesthetized with diethyl ether and identified using standard morpho-taxonomic keys [17, 18]. Morphological features used for identification were head, proboscis, thorax, legs, wings and palpi. The mosquitoes were individually placed in 1.5 mL microfuge tubes. The tubes were labelled with specific codes, sealed and placed in separate boxes designated to capture information on origin of the mosquito (including the time, place, and exact trap they came in). The samples were preserved at −80 °C for further analysis.

### DNA extraction

Mosquitoes collected from May to July 2013 were not separated prior to DNA extraction. The rest of the mosquitoes were dissected into two parts, head/thorax (H) and abdomen (A) by making a sharp cut between the thorax and abdomen. The legs and wings were detached from the main body and stored separately at −80 °C. Genomic DNA from whole mosquitoes or individual parts (H and A) of each anopheline mosquito was extracted using QIAamp DNA Mini Kits (Qiagen, Germany). Isolated DNA, eluted in 100 µL nuclease free water (Ambion) was then stored at −80 °C for further use. DNA extracted from individual head and abdomen parts was used as the template for the PCR reaction, except for mosquitoes collected from May to July 2013. In these cases, DNA extracted from the whole mosquito was used as a template.

### Blood meal analysis

To determine the potential anthropophagic nature of the mosquitoes, abdomens were screened for the presence of human and bovine blood by PCR using specific primers derived from ribosomal RNA intergenic spacer sequence of *Homo sapiens* and mitochondrial DNA (mtDNA) of *Bos taurus* respectively (Table 1). The PCR conditions were: initial denaturation at 95 °C for 4 min followed by 35 cycles of denaturation at 95 °C for 30 s, annealing at 50 °C for 30 s, extension at 72 °C for 1 min and final extension at 72 °C for 8 min [19]. *Anopheles stephensi* and *An. subpictus* were also screened for bovine blood using specific primers by PCR as described by Corona et al. [20]. The PCR conditions were: initial denaturation at 95 °C for 2 min; 10 cycles of denaturation at 94 °C for 1 min, annealing at 58 °C for 1 min, extension at 72 °C for 1 min 30 s; 20 cycles of denaturation at 90 °C for 1 min, annealing at 58 °C for 1 min, extension at 72 °C for 1 min 30 s; and final extension at 72 °C for 10 min.

**Table 1:** Primers for detection of human blood, bovine blood and malaria parasites

Species	Target gene	Primer name	Sequence (5′–3′)	PCR product size (bp)	References
*Homo sapiens* (human)	rDNA	HUM1	CGAGAGTTC//TCTGGAAGAATTGA	519	Mohanty et al. 2007 [16]
		HUM2	TGATAGCCTGGAAGTGACAAAAT		
Bovine *Bos taurus*	mtDNA	B1	CATCATAGCAATTGCCATAGTCC	165	Corona et al. 2007 [20]
		B2	GTACTAGTAGTATTAGAGCTAGAATTAG		
*Plasmodium* (genus-wide)	18S rRNA	rPLU5	CCTGTTGTTGCCTTAAACTTC	1100	Johnston et al. 2006 [18]
		rPLU6	TTAAAATTGTTGCAGTTAAAACG		
*P. falciparum* (species specific)	18S rRNA	rFAL1	TTAAACTGGTTTGGGAAAACCAAATATATT	205	
		rFAL2	ACACAATGAACTCAATCATGACTACCCGTC		
*P. vivax* (species specific)	18S rRNA	rVIV1	CGCTTCTAGCTTAATCCACATAACTGATAC	120	
		rVIV2	ACTTCCAAGCCGAAGCAAAGAAAGTCCTTA		

### Sibling species analysis

The sibling species identification of *An. subpictus* using allele-specific PCR was carried out using the gene specific ITS2 region and confirmed by sequence analysis. The PCR conditions were: initial denaturation at 95 °C for 4 min, followed by 35 cycles of denaturation at 95 °C for 30 s, annealing at 50 °C for 30 s, extension at 72 °C for 1 min, and final extension at 72 °C for 8 min.

### Mosquito infection status

The mosquito samples were screened for *Plasmodium* by PCR, initially using genus specific universal primers (28S rDNA specific universal primer set) (Table 1) [21, 22]. The following mixture was subjected to *Plasmodium* universal PCR: 1.5 µL 10X PCR buffer (500 mM KCl, 200 mM Tris-HCl), 1.0 µL of 50 mM MgCl_2_, 2.5 µL of 2.5 mM dNTPs each (GeNei, Bangalore), 0.5 µL oligonucleotide primers (Eurofins Genomics India Pvt Ltd) 20 pM/µL each, 0.5 µL (5 units/µL) of *Taq* polymerase (Invitrogen), 3.5 µL of nuclease free water (Ambion) and 15 µL of template DNA in a final volume of 25 µL. The PCR conditions were set as follows: initial denaturation at 94 °C for 5 min followed by 40 cycles of denaturation at 94 °C for 30 s, annealing at 55 °C for 1 min, extension at 72 °C for 2 min, and final extension at 72 °C for 5 min.

### Species-specific nested PCR

In order to identify the species of *Plasmodium* in the infected mosquitoes, the initial amplified PCR product was generated using genus specific primers. This was then purified using Qiagen PCR purification kits and subjected to nested PCR using species specific primers [19, 21, 22]. Nested PCR was performed using the following mixture: 2.0 µL 10X PCR buffer (500 mM KCl, 200 mM Tris-HCl), 1.0 µL (50 mM MgCl_2_), 2.0 µL (2.5 mM of each dNTPs), 0.5 µL of each primer (20 pM/µL), 0.5 µL (5 units/µL) of *Taq* polymerase, 8.5 µL of nuclease free water and 10 µL of PCR product in a total volume of 25 µL. The PCR conditions were: initial denaturation at 94 °C for 5 min followed by 40 cycles of denaturation at 94 °C for 1 min, annealing at 55 °C for 2 min, extension at 72 °C for 2 min, and final extension at 72 °C for 5 min.

### Data and sample management

REDCap electronic data management tools were utilized to record all information related to trap placement, collection and mosquito identification [23]. A FreezerPro database was used to manage mosquito and mosquito derivative storage. Data recorded in both systems were available to MESA-ICEMR partner labs in both India and the USA in real-time.

## Results

### Anophelines collected with CDC traps

A broad and unbiased approach was taken to determine which anopheline species in Goa may currently be transmitting human malaria. In all 23,782 mosquitoes belonging to 27 species were collected including 16,201 females and 7586 males. A total of 1375 anopheline mosquito specimens were caught in 1036 traps deployed during the study period. This collection included ten *Anopheles* species. One of the two dominant anopheline species was the well-studied urban and semi-urban vector *An. stephensi* (334, 24.3 %). Another abundant vector in the traps was *An. subpictus* (501, 36.4 %), actually out numbering *An. stephensi.* Together, the two species constituted 60.7 % of the total anophelines trapped. There were eight other less abundant anopheline mosquito species in the traps, including 363 *An. jamesii* (26.4 %), 66 *An. vagus* (4.8 %), 57 *An. annularis* (4.1 %), 36 *An. nigerrimus* (2.6 %), 9 *An. barbirostris* (0.6 %), 7 *An. tessellates* (0.5 %), and 1 each of *An. umbrosus* and *An. karwari*.

### Seasonality of the dominant anophelines

**Fig. 1:**
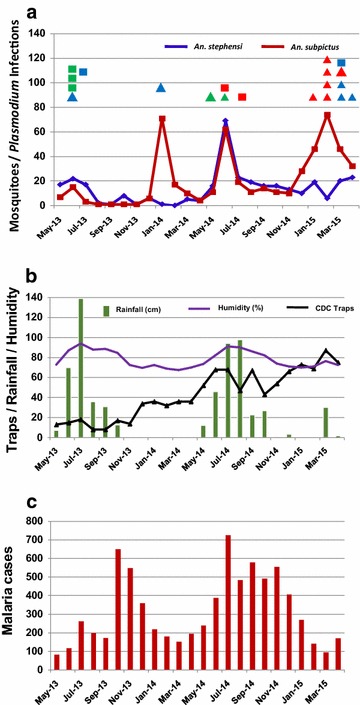
Relationship between vector buildup, infected mosquitoes, rainfall in Goa, and numbers of malaria patient cases in Goa. **a** *Anopheles stephensi* and *Anopheles subpictus* prevalence from May 2013 to April 2015 (*blue* and *red lines*, respectively) and times of mosquito *Plasmodium* infections from May 2013 to April 2015 (*squares* and *triangles* *above the lines*). Each *spot depicts* one *Plasmodium* infection/mosquito in different months. The colour key is as follows: *Blue square*: *An. stephensi* (*Pv* + *Pf*), *Green square*: *An. stephensi* (*Pv*), *Red square*: *An. stephensi* (*Pf*), *Blue triangle*: *An. subpictus* (*Pv* + *Pf*), *Green triangle*: *An. subpictus* (*Pv*), *Red triangle*: *An. subpictus* (*Pf*). **b** Seasonal variation in traps placed (*purple line with triangles*), rainfall (*green bars*), humidity (*plain purple line*). The scale on the left applies to all three measurements (number of traps, rainfall in cm, and humidity in % value). **c** Annual distribution of malaria cases at clinics and hospitals in Goa

Not only were the absolute numbers of captured *An. subpictus* higher than those of *An. stephensi*, but *An. subpictus* showed a wider annual prevalence. As shown in Fig. 1a (red line), *An. subpictus* mosquitoes were captured in high numbers from May to July (117/501, 23.3 %), but prevalence continued later including a prominent peak between December and April (334/501, 66.7 %). For reference, the rainier months in Goa are from May to October and the dry period is from November to April (Fig. 1b, green bars). As expected and as shown in Fig. 1a (blue line), the conventional vector *An. stephensi* collections were most abundant during the monsoon months of May to October (226/334, 67.6 %), but the numbers of *An. stephensi* decreased during the drier months with the 6 months of November to April yielding 108 *An. stephensi* (32.4 %).

### Blood meal analysis of the anophelines

**Fig. 2:**
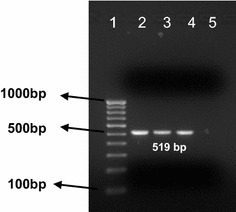
Demonstration of human blood in representative mosquitoes. PCR amplification of ribosomal RNA intergenic spacer sequence (rDNA) of *H. sapiens*: *Lane 1* 100 bp DNA ladder; *Lane 2* and *Lane 3* *An. stephensi* (519 bp; sample ID, ST65 and ST66); *Lane 4* *An. subpictus* (519 bp; sample ID, SP28); *Lane 5* Negative control (nuclease free water)

It was important to test whether *An. subpictus,* as expected for *An. stephensi*, was biting human hosts. Indeed, when tested for human blood with PCR (Fig. 2), the two most prevalent *Anopheles* species were positive for human blood at comparable frequencies.

Out of 334 *An. stephensi*, 100 were positive for human blood (29.9 %), but none for bovine blood. Of these, 15.4 % *An. stephensi* assayed were positive for human blood during the monsoon phase (May–October) and 14.5 % were positive during the dry period (November–April), showing no seasonal variation. Out of 501 *An. subpictus,* 135 were positive for human blood (26.9 %) and only six were positive for bovine blood (1.2 %). In contrast with *An. stephensi*, 2.4 % *An. subpictus* assayed were positive for human blood during the monsoon phase and 24.1 % were positive during the dry period, showing significant seasonality.

Three additional anopheline species were positive for human blood: 80 *An. jamesii* (21.9 %), 15 *An. annularis* (25.9 %), and 6 *An. nigerrimus* (17.1 %). None of the remaining five *Anopheles* species tested positive for human blood.

### Plasmodium infections

**Fig. 3:**
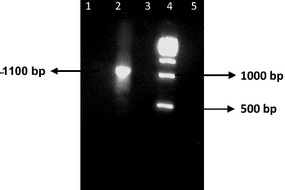
Demonstration of successful PCR amplification of *Plasmodium* DNA using universal primers to detect an infection. *Lane 1* & *3* (Negative controls); *Lane 5* negative control (Nuclease free water); *Lane 2* positive (1100 bp; Sample ID, SP328); *Lane 4* (500 bp DNA ladder)

**Fig. 4:**
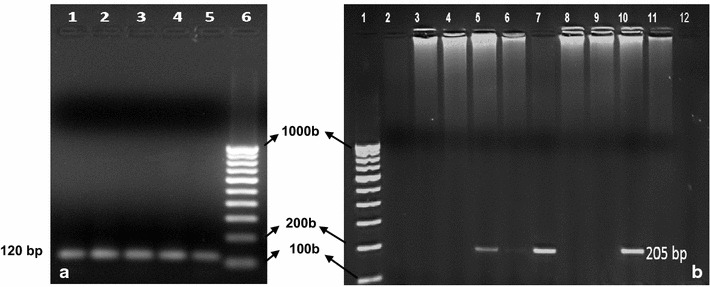
Demonstration of representative nested PCR amplification to detect *P. vivax* (**a**) and *P. falciparum* (**b**): **a** *Lane 1,2,3,4* *An stephensi* (Mosquito sample ID, ST24, ST31, ST32, ST54) positive for *P. vivax*; *Lane 5* *An. subpictus* positive for *P. vivax* (SP12); *Lane 6* 100 bp DNA ladder **b** *Lane 1* 100 bp DNA ladder, *Lane 2,3,4,6,8,9,11,12* (Negative, Sample ID, SP315, SP322, SP325, SP369, SP386, SP389, SP392, SP 399). *Lane 5, 7* are positive for *An. subpictus* (SP328, SP371) and *Lane 10* is positive for *P. falciparum* in *An. stephensi* (ST298)

To determine which anophelines in the collection harboured human malaria parasites, diagnostic PCR was conducted on each of the anopheline mosquitoes. In the case of *An. subpictus, Plasmodium* infection was seen in 2.8 % (14/501) of specimens as revealed by PCR amplification of a 1100 bp DNA fragment using *Plasmodium* specific universal primers (Fig. 3). In addition, nested PCR was used to identify the species of *Plasmodium* causing infection (Fig. 4). Out of these 14 *An. subpictus* positive for the *Plasmodium* genus, two (0.6 %) showed *P. vivax* mono-infection, six (1.2 %) showed *P. falciparum* mono-infection, and another six (1.2 %) harboured a mixture of *P. vivax* and *P. falciparum* confirmed by nested PCR (Table 2). The infectivity rate based on salivary gland positivity in *An. subpictus* (0.6 %) was comparable to that of *An. stephensi.*

**Table 2:** Detection of *Plasmodium* species in *Anopheles subpictus* and in *Anopheles stephensi*

Vector species	No. *Plasmodium* +ve/assayed	Parts of mosquito tested	Number tested	Only *Pv*	Only *Pf*	*Pv* + *Pf*	Total positive	Infection/infectivity rate (%)
*An. subpictus*	14/501	Whole	25	0	0	1	1	2.8^b^
		Abdomen	476	1	6	5 (2^a^)	12 (2^a^)	
		Head	476	1	0	2^a^	3 (2^a^)	0.6^c^
*An. stephensi*	7/334	Whole	56	3	0	1	4	2.1^b^
		Abdomen	278	0	2 (1^a^)	1^a^	3 (2^a^)	
		Head	278	0	1^a^	1^a^	2^a^	0.6^c^

*Pv* *P. vivax*; *Pf* *P. falciparum*

^a^Indicates same mosquito tested positive for both head and abdomen hence counted once

^b^Indicates infection rate

^c^Indicates infectivity rate

For comparison, *Plasmodium* infection was seen in 2.1 % (7/334) of the traditional vector *An. stephensi* as revealed by PCR amplification of an 1100 bp DNA fragment using *Plasmodium* specific universal primers (Fig. 3). In addition, three out of the seven infected *An. stephensi* were positive for *P. vivax* alone (120 bp amplicon), two (0.6 %) had *P. falciparum* alone (205 bp band size) and another two (0.6 %) contained both *P. vivax* and *P. falciparum* (Table 2; Fig. 4). Finally, anthropophily of the two vectors that were positive for human malaria parasites were cross-checked. Seven of the 14 *Plasmodium* positive *An. subpictus* also showed a positive reaction for human blood as did five of the seven *An. stephensi* positive for *Plasmodium*.

A preliminary study using allele-specific PCR suggests that both sibling species A and B of *An. subpictus* occur in equal proportions. Three *An. subpictus* sibling species ‘B’ were *Plasmodium* positive in the salivary glands, suggesting a role in malaria transmission in Goa. The role of other *An. subpictus* sibling species in malaria transmission needs to be further investigated.

### Temporal distribution of vector infections in urban Goa

The time frames for *Anopheles* species with *P. falciparum* and *P. vivax* infections allowed first glimpses of when each mosquito species was most active at transmitting malaria. The conventional vector, *An. stephensi*, was most active as a vector from May to July. Unlike *An. stephensi, An. subpictus* were found positive for malaria parasites from December to April as well as May to July (Table 3; Fig. 1a). This may help explain ongoing malaria cases being reported during these months at local clinics and hospitals (Fig. 1c).

**Table 3:** Transmission periods assessed by times of positive specimens in the CDC trap collections

Vector	*P. vivax*	*P. falciparum*	*P. vivax* + *P. falciparum*
*An. subpictus*	May 1	January 1	January 2
	June 1	February 4	March 2
		March 1	April 1
			June 1
*An. stephensi*	June 3	June 1	March 1
		August 1	July 1

The seasonal analysis of anthropophagic index of these two vector species and their specimens positive for human *Plasmodium* showed good correlation in the case of *An. subpictus*. While 3/501 (0.6 %) *An. subpictus* were positive for human *Plasmodium* species during the pre-rainy season, its anthropophagic index was found to be 2.8 %. In contrast, 121/501 (24.1 %) *An. subpictus* were positive for human *Plasmodium* species during the dry period while its infection rate was 2.2 %. In contrast, during the rainy season 6/334 (1.8 %) *An. stephensi* tested *Plasmodium* positive while its anthropophagic index was 14.4 %. In the dry period, in spite of a 15.6 % human blood index, the *Plasmodium* infection rate was only 0.3 %.

### Geographic distribution of vector infections in urban Goa

The presence of *An. subpictus* and its infectivity was not restricted to one specific part of urban Goa. In the present study, 343, 218, 124 and 150 CDC traps were placed in Panaji, Candolim, Porvorim and Margao, respectively, for a total of 1036 traps (Table 4). There was an even distribution of infected versus uninfected mosquitoes in traps between the different towns. The *Plasmodium* infection in *An. subpictus* and *An. stephensi,* were 8 (2.3 %), 4 (1.8 %), 4 (3.2 %), and 5 (3.3 %), respectively, in these four locations. On average, 2.0 % of the CDC traps captured infected mosquitoes.

**Table 4:** *Anopheles* *subpictus* and *Anopheles stephensi* collections and their infection status at multiple sites in Goa

Locality in Goa	No. of traps	No. of *Anopheles*			Mean *Anopheles* vectors/trap	No. *Anopheles* *Plasmodium*+ve (IR %)	% Trap with infected vectors
		*stephensi*	*subpictus*	Total			
Panaji +	328	142	201	343	1.04	8 (2.3)	2.4
Candolim	245	85	133	218	0.88	4 (1.8)	1.6
Porvorim	176	47	77	124	0.70	4 (3.2)	2.3
Margao	287	60	90	150	0.52	5 (3.3)	1.7
Total	1036	334	501	835	0.8	21 (2.5)	2.0

*Panaji+* Panaji and surroundings, *IR* combined infection rate in vectors

## Discussion

Traditional views of major urban malaria vectors in western India do not include *An. subpictus.* While *An. subpictus* infected with *P. falciparum* and *P. vivax* is new in Western India, and in Goa in particular, *An. subpictus* has been reported as a malaria vector in Sri Lanka, Eastern and Central India, Maldives and several other parts of South and South East Asia [24–31].

In the present open-ended study, CDC traps were continuously deployed in four malaria endemic towns of Goa over a period of two years. *An. subpictus* was found to harbour *Plasmodium* alongside *An. stephensi* in Goa. Salivary gland positivity further suggested that *An. subpictus* was capable of propagating both *P. vivax* and *P. falciparum* in humans in Goa. The present collection showed one of the highest reported figures for *An. subpictus* testing positive for human blood in an urban area [28]. Furthermore, *An. subpictus* showed an infection rate of nearly 2.8 % and an infectivity rate of 0.6 % (Table 2), which is comparable to earlier findings [27]. Other known malaria vectors in non-urban and forested areas of Goa, *An. culicifacies* and *An. fluviatilis*, were not caught in CDC traps, confirming earlier findings [6, 8, 9].

It was surprising and important to note that not only was *An. subpictus* the predominant anopheline mosquito, but that it also had two prevalence peaks during the year. *Anopheles subpictus* showed a wider window of transmission from December to April and then again from May to July, overlapping with the *An. stephensi* peak transmission. It appears that the two vectors work in tandem to sustain continual malaria transmission throughout the year. Without *An. subpictus*, *An. stephensi* may not be able to sustain transmission through the drier months itself.

An interesting question is how *An. subpictus* is better able to propagate than *An. stephensi* during the dry season of December to April. While *An. stephensi* breeds mainly in manmade habitats in Goa such as curing waters, masonry tanks, terraces, lintels, overhead tanks and wells, *An. subpictus* additionally breeds in a variety of natural puddles, pools, ponds, pit wells, rice paddies and backwaters [5, 6]. This wider choice of breeding habitat may give *An. subpictus* one advantage over *An. stephensi*. Detailed work on the south coast of Java showed the salt water sibling species ‘B’ of *An. subpictus* complex positive for sporozoite infection in salivary glands [32, 33]. The fact that *An. subpictus* can breed in fresh water as well as salt water systems, where it can tolerate a very high degree of salinity, may have important implications for its ability to breed year round [4].

One important outcome of this study is that continual, open-ended surveys of all potential vectors of human malaria transmission can be an effective monitoring tool to guide control strategies, particularly in urban areas, and may help identify conventionally overlooked vectors.

## Conclusions

The present study shows the presence of *P. vivax* and *P. falciparum* in both salivary glands and mid-guts of *An. subpictus* in Goa, India. The study highlights that *An. subpictus* is more prevalent than the recognized primary urban vector, *An. stephensi* in the coastal region of Goa. There is high probability that *An. subpictus* contributes to the propagation of malaria in urban Goa especially during the post-monsoon period when *An. stephensi* numbers decline. Given that *An. subpictus* is present throughout India and Asia and can breed year-round in coastal areas, monitoring and further study of *An. subpictus* should be an integral part of national urban vector control measures in Asia.

## Abbreviations

CDC: Centres for Disease Control and Prevention
CSP: circumsporozoite protein
DDT: dichlorodiphenyl trichloroethane
DNA: deoxynucleic acid
ELISA: enzyme linked immune sorbent assay
HBI: human blood index
ICEMR: International Centre of Excellence for Malaria Research
MESA: Malaria Evolution in South Asia
NIAID: National Institute of Allergy and Infectious Diseases
NVBDCP: National Vector Borne Diseases Control Programme
PCR: polymerase chain reaction
RNA: ribose nucleic acid
USA: United States of America
UV: ultraviolet
WHO: World Health Organization
